# Assessment of *PALB2* as a Candidate Melanoma Susceptibility Gene

**DOI:** 10.1371/journal.pone.0100683

**Published:** 2014-06-20

**Authors:** Lauren G. Aoude, Mai Xu, Zhen Zhen Zhao, Michael Kovacs, Jane M. Palmer, Peter Johansson, Judith Symmons, Jeffrey M. Trent, Nicholas G. Martin, Grant W. Montgomery, Kevin M. Brown, Nicholas K. Hayward

**Affiliations:** 1 QIMR Berghofer Medical Research Institute, Brisbane, QLD, Australia; 2 University of Queensland, Brisbane, QLD, Australia; 3 National Cancer Institute, Bethesda, Maryland, United States of America; 4 Translational Genomics Research Institute, Phoenix, Arizona, United States of America; CNR, Italy

## Abstract

Partner and localizer of BRCA2 (PALB2) interacts with BRCA2 to enable double strand break repair through homologous recombination. Similar to *BRCA2*, germline mutations in *PALB2* have been shown to predispose to Fanconi anaemia as well as pancreatic and breast cancer. The PALB2/BRCA2 protein interaction, as well as the increased melanoma risk observed in families harbouring *BRCA2* mutations, makes *PALB2* a candidate for melanoma susceptibility. In order to assess *PALB2* as a melanoma predisposition gene, we sequenced the entire protein-coding sequence of *PALB2* in probands from 182 melanoma families lacking pathogenic mutations in known high penetrance melanoma susceptibility genes: *CDKN2A, CDK4,* and *BAP1*. In addition, we interrogated whole-genome and exome data from another 19 kindreds with a strong family history of melanoma for deleterious mutations in *PALB2*. Here we report a rare known deleterious *PALB2* mutation (rs118203998) causing a premature truncation of the protein (p.Y1183X) in an individual who had developed four different cancer types, including melanoma. Three other family members affected with melanoma did not carry the variant. Overall our data do not support a case for *PALB2* being associated with melanoma predisposition.

## Introduction

Familial melanoma represents approximately 5–10% of all cutaneous malignant melanoma (CMM) cases and it is estimated that approximately 40% of familial cases can be attributed to known high penetrance genes. The cause of increased risk of melanoma development in the remaining families is largely unknown but might be due to a combination of low or medium penetrance gene mutations or to rare high penetrance mutations that are as yet undiscovered. A plausible candidate that might contribute to the melanoma risk landscape in certain individuals is partner and localizer of *BRCA2* (*PALB2*). PALB2 has a role in DNA repair, which it does by binding to BRCA1 and BRCA2 to facilitate homologous recombination for repair of double-strand breaks. It also has a role in facilitating a DNA checkpoint response and disruption to this complex can lead to instability of the DNA damage response pathway [1]. Germline mutations in its interacting partner BRCA2 are known to predispose to cancers of the breast, ovary and pancreas, as well as confer a moderate increased risk of CMM (relative risk of 2.6 [2], [3]), suggesting by association that PALB2 may also play a role in tumour development in these cancer types.

There have been several diseases attributed to germline mutation in *PALB2*, the first reports were in Fanconi anemia (FA) patients with the FA-N subtype [4], [5]. Carriers were affected by bi-allelic mutation in *PALB2* that led to the onset of early childhood cancers, along with other FA disease traits including growth retardation and congenital malformation. Looking at the family histories of these cases, 4/8 families had a history of breast cancer, suggesting that like *BRCA2*, mono-allelic mutations in *PALB2* may lead to the development of breast cancer.

From this observation, germline *PALB2* mutations were subsequently shown to be associated with breast cancer risk [6], [7]. Rahman and colleagues investigated a cohort of 923 breast cancer cases of which 10 individuals presented with mono-allelic truncating *PALB2* mutations, while a set of 1,084 controls harboured no truncating mutations. Segregation analysis showed that in approximately half of these families the variants did not completely co-segregate with disease, suggesting that *PALB2* acts as a medium-penetrance rather than high-penetrance gene for breast cancer risk [6]. A study published at the same time by Erkko et al., reported a founder mutation, c.1592delT, present in 0.2% of the Finnish population, but 1% of breast cancer cases unselected for family history and 2.7% of families with multiple cases of breast and/or ovarian cancer [7]. This shows a clear role for *PALB2* in breast cancer development. Since these initial reports, there has been a wide array of population-based studies describing the prevalence of *PALB2* in breast cancer cases with incidence ranging from 0.5% to 2.6% [8]–[18].

An association between germline *PALB2* mutation and increased pancreatic cancer risk has also been established. After identifying bi-allelic inactivation of *PALB2* in a tumour from a familial pancreatic cancer patient, Jones and colleagues investigated germline *PALB2* variation in 96 probands with a family history of pancreatic cancer and identified truncating mutations in three individuals [19]. In another study, the screening of 254 pancreatic cancer cases, including 101 with a family history of the disease, led to the report of a 6.7 kb deletion of *PALB2* in an individual with both breast and pancreatic cancer. This was the only conclusively inactivating mutation found in this sample set [20]. A study looking at European familial pancreatic cancer discovered truncating mutations in 3/81 cases, all of which were from families with cases of breast cancer, suggesting that these mutations may preferentially occur in families with both of these cancer types [21]. Consistent with this, another study reported that 4.8% of families with both cancer types harboured truncating *PALB2* mutations [22], higher than the rate previously reported in studies investigating breast cancer alone. Although a study that followed on from this looked at 77 families with breast cancer, pancreatic cancer, or a combination of both, but found no mutations [23]. Because *PALB2* germline truncating mutations are relatively rare, the full spectrum of cancer predisposition associated with these mutations has yet to be fully characterized. This is clear from the differing reports relating PALB2 to breast and pancreatic cancer development.

Multiple studies have reported melanoma in families harbouring inactivating *PALB2* mutations, including individuals with diagnoses of both melanoma and breast or pancreatic cancer [6] [24]. These data implicate *PALB2* as a possible melanoma susceptibility gene, although Sabbaghian and colleagues found no association between germline *PALB2* mutation and CMM risk in a screen of 53 probands from multi-case *CDNK2*A mutation-negative melanoma families, and Yang failed to identify *PALB2* mutations in 23 CMM families that are *CDKN2A* mutation-positive and contain a subset of families (n = 11) with pancreatic cancer also [25]. Given the small size of these studies, the association between *PALB2* and melanoma risk remains unclear. We therefore sought to determine the incidence of germline *PALB2* mutations in a larger series of 201 *CDKN2A* and *CDK4* mutation-negative melanoma families, including 63 with confirmed cases of breast, pancreatic, or multiple other types of cancer.

## Methods

### Ethics

Written consent was obtained from each participant in this study. Ethics approval was obtained from the QIMR Berghofer Human Research Ethics Committee (HREC).

### Sample collection

Samples were ascertained as part of the Queensland Familial Melanoma Project (QFMP), a population based study of melanoma in Queensland, Australia [26]. Genomic DNA was extracted from whole blood using standard salting out methods. In some instances DNA was extracted from transformed lymphoblastoid cell lines.

### Samples

Selection criteria for inclusion of families were those with: CMM plus breast and/or pancreatic cancer (n = 52); individuals who had developed three or more different cancer types, where CMM was one of the cancers (n = 3); a minimum of three CMM cases (n = 127). A total of 182 families met these criteria. No additional criteria relating to age of onset of the cancers, or degree of relationship between affected members were imposed. All families have previously been shown to lack pathogenic mutations in known high risk melanoma susceptibility genes, *CDKN2A*, *CDK4* and *BAP1* [*27]*, [28]. In the instance where multiple DNA samples were available for sequencing, the youngest available CMM case was chosen as the proband for each family. Where affected individuals within a family were of a comparable age and an individual presented with multiple primary melanomas, they were then selected as the proband.

In addition to this, whole-genome and whole-exome sequencing data from some QFMP families (n = 19) was interrogated. These families were selected for sequencing as they had a minimum of three affected members. Within this group, 16/19 families had cases with multiple primary melanoma, and 8/19 families had occurrences of breast cancer. Six families also included individuals that had developed three different cancer types. A total of 24 exomes and 15 genomes were interrogated, with up to 3 cases sequenced within a family. None of the cases sequenced carried deleterious variants in breast cancer susceptibility genes *BRCA1* or *BRCA2*.

### Sanger sequencing

In order to look for pathogenic mutations in *PALB2*, Sanger sequencing was used to screen the 13 exons as well the exon boundaries of the 182 selected probands for protein altering variants. The complete list of M13-tagged primers and sequencing methods can be found in Methods S1.

### Whole-genome and exome sequencing

Whole-genome sequencing for 12 samples and exome sequencing for 25 samples was performed using the Illumina Hiseq 2000 platform combined with the Agilent SureSelect Human All Exon V4+UTRs enrichment kits (Methods S1). 100 bp paired-end reads were generated with samples having a mean coverage of 96X. A further three genomes were sequenced by Complete Genomics. Using the BWA alignment algorithm, the sequence output was mapped to the UCSC human genome reference build 19 [29]. SNPs and indels were detected using bcftools and SAMtools mpileup with disabled BAQ computation [30]. Each sample had on average 90,000–100,000 variants compared to the human genome reference sequence. Variants were filtered for stringency using a quality score (>40), alternate reads (>2 and >20% of all reads at a given position). Variants from next-generation sequencing data sets were validated using Sanger sequencing methods. Whole-genome and exome sequencing data may be made available for research purposes upon request to the authors.

### Copy number analysis at the *PALB2* locus

To assess the possibility that some probands may have partial or complete gene deletions of *PALB2* we interrogated the whole-genome or exome data from those patients where data was available. Briefly, to normalise for different coverage per sample, the number of reads for each exon was divided by the number of reads across the entire sample. The median value for each exon was then calculated across all samples. This value was used to estimate copy-number variation per exon of *PALB2.* In instances where a copy number value for a given exon in an individual was estimated to be less than half of the median value across all patients, quantitative PCR using SYBR Green and exon-specific primers was then used to assess the validity of the bioinformatics output.

### Case-control analysis

All protein-changing variants that were identified by Sanger sequencing and exome sequencing were analysed in a case-control set derived from two Australian studies to determine whether they might be low to medium penetrance CMM predisposition variants. A Sequenom iPLEX was run on 3320 individuals which included 1,690 probands derived from the QFMP [26]. These individuals were sampled from Queensland, Australia and include both cases with a first degree relative with CMM (n = 1551); and also sporadic cases with no family history of melanoma (n = 139). The cases included individuals with a wide spectrum of age of disease onset that ranges from childhood disease to late onset melanoma.

The control sample (n = 1630) were parents of twins ascertained as part of the Brisbane Twin Naevus Study [31]. They were asked to self report and had no history of melanoma at the time of sample collection.

In order to analyse the significance of observed protein altering variants found through sequencing methods, a chi-squared test was used to compare the cases and controls.

## Results

We sequenced the entire protein coding sequence of *PALB2* in the panel of CMM cases described above. We identified eight missense variants, four synonymous variants and one nonsense variant. Of these, 11 were present in NHLBI Exome Sequencing Project (ESP6500) [32], dbSNP or the 1000 Genomes Project (see Table 1 for a full list of variants). No proband for which whole-genome (n = 12) or exome (n = 25) sequence data were available was found to carry a partial or complete deletion of *PALB2*.

**Table 1:** *PALB2* variants detected through Sanger sequencing and exome sequencing.

Location	proteinchange	nucleotidechange	rs ID	freq. of genotypein CMM probands(n = 201)	MAF in CMMprobands	MAF in ESP6500*(n = 4300)	SIFTprediction	protein domain function	reference
chr16∶23647635	p.V78I	c.232G>A	–	1/201	0.002	na	tolerated	interacts with BRCA1 & RAD51;required for oligomerization	[33]
chr16∶23646857	p.L337S	c.1010T>C	rs45494092	8/201	0.020	0.020	tolerated		[17], [18], [20], [24], [33], [35], [36]
chr16∶23646673	p.V398V	c.1194G>A	rs61755173	2/201	0.005	0.002	na		[12]
chr16∶23646295	p.S524S	c.1572A>G	rs45472400	3/201	0.007	0.005	na		[12], [18]
chr16∶23646191	p.Q559R	c.1676A>G	rs152451	33/201	0.082	0.091	tolerated		[17], [18], [20], [24], [33], [35]–[37]
chr16∶23641461	p.E672Q	c.2014G>C	rs45532440	13/201	0.032	0.031	tolerated		[17], [18],
chr16∶23637715	p.P864S	c.2590C>T	rs45568339	3/201	0.007	0.003	tolerated	WD1; interacts with BRCA2 & RAD51	[17], [18], [33], [35], [38]
chr16∶23635370	p.V932M	c.2794G>A	rs45624036	2/201	0.005	0.005	tolerated	WD2; interacts with BRCA2 & RAD51	[20], [33], [35]
chr16∶23635348	p.L939W	c.2816T>G	rs45478192	2/201	0.005	0.002	damaging	WD2; interacts with BRCA2 & RAD51	[33], [35]
chr16∶23634293	p.G998E	c.2993G>A	rs45551636	12/201	0.030	0.023	damaging	WD3; interacts with BRCA2 & RAD51	[17], [18], [20], [24], [33], [35], [36]
chr1 6:23619235	p.T1100T	c.3300T>G	rs45516100	11/201	0.027	0.031	na	WD4; interacts with BRCA2 & RAD51	[18], [20], [35], [36]
chr16∶23614846	p.S1165S	c.3495G>A	rs45439097	1/201	0.002	0.001	na	WD7; interacts with BRCA2 & RAD51	[35]
chr16∶23614792	p.Y1183X	c.3549C>G	rs118203998	1/201	0.002	na	na	WD7; interacts with BRCA2 & RAD51	[5], [6], [8], [34]

*European American population.

na is not available.

**Figure 1:**
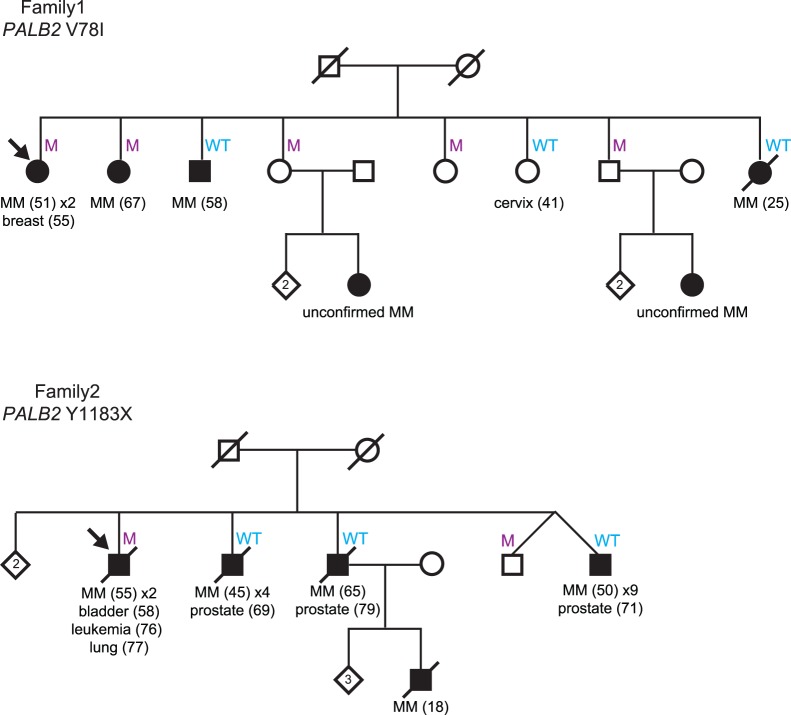
Co-segregation analysis of *PALB2* variants in two high-risk CMM families. Individuals that have melanoma (MM) are represented by black circles (female) and black boxes (male). The age of diagnosis of each cancer is indicated in brackets. A line through a symbol indicates that the person is deceased. Individuals carrying a *PALB2* mutation are indicated by an ‘M’, while those wild-type for the variant are indicated by ‘WT’. Other cancer types are also indicated on the pedigree. Unaffected siblings are represented by a diamond with the number indicating the number of siblings. The arrow indicates the proband in each family.

A missense variant, p.V78I (c.G432A, NCBI accession NM_024675), not listed in dbSNP but reported previously in a study of families with breast/ovarian and pancreatic cancers [33], was identified via Sanger sequencing and found in a female who presented with two primary CMM, at ages 51 and 71, and breast cancer at age 55. Co-segregation analysis showed incomplete segregation with disease in the remainder of the family (Figure 1). Only two of the four individuals affected with CMM in this family carry *PALB2* p.V78I, the second carrier being a sibling who had developed CMM at age 67. A protein-truncating variant, p.Y1183X, was found in a five case CMM family that was analysed via exome sequencing. This variant, rs118203998, was originally identified in a breast cancer family by Rahman and colleagues [6] and has since been reported by several other groups in patients diagnosed with breast cancer, pancreatic cancer, and Fanconi anaemia [5], [6], [8], [34]. The individual carrying this mutation in our study has been diagnosed with four different primary cancer types, including melanoma (twice, initially diagnosed at age 55), bladder cancer (at age 58), leukaemia (at age 76), and non-small cell lung cancer (at age 77). This variant did not co-segregate with melanoma in this family as no other affected family member was found to be a carrier.

Genotyping a collection of 1,690 CMM probands, as well as 1630 Australian population controls, showed that *PALB2* p.Y1183X was not observed in any other individual. This is consistent with results found by Rahman [6]. This variant is also not seen in the 1000 Genomes Project, nor is it seen in any population of the ESP6500, and is listed in dbSNP as having unknown population frequency.

The p.V78I was observed in two controls. Personal history of non-melanoma cancers was not collected at the time of the study setup. It is unclear whether these individuals may have been affected by any other form cancer. The other seven rare missense variants we identified in the family collection were found at low frequency in the case-control panel (Table 2). None were significantly over-represented in melanoma cases.

**Table 2:** Case-control analysis on non-synonymous *PALB2* variants.

location	protein change	nucleotide change	rs ID	Frequency in cases (n = 1690)		Frequency in controls (n = 1630)		chi-square	p-value
				het carriers	hom carriers	het carriers	hom carriers		
Exon 4	p.V78I	c.232G>A	–	1	0	0	0	–	–
Exon 4	p.L337S	c. 1010T>C	rs45494092	79	2	71	0	0.565	0.452
Exon 4	p.Q559R	c. 1676A>G	rs152451	289	12	243	10	2.981	0.084
Exon 5	p.E672Q	c. 2014G>C	rs45532440	105	0	94	1	0.148	0.700
Exon 7	p.P864S	c. 2590C>T	rs45568339	8	0	18	0	4.202	0.040
Exon 8	p.V932M	c. 2794G>A	rs45624036	13	0	13	0	0.009	0.924
Exon 8	p.L939W	c. 2816T>G	rs45478192	6	0	9	0	0.715	0.398
Exon 9	p.G998E	c. 2993G>A	rs45551636	73	0	66	0	0.148	0.700
Exon 13	p.Y1183X	c. 3549C>G	rs118203998	1	0	0	0	–	–

## Discussion

To assess the contribution of *PALB2* to melanoma predisposition we sequenced the protein-coding region of *PALB2* in probands from 201 melanoma families lacking pathogenic mutations in know melanoma susceptibility genes. This is the largest study reported to date to assess the relationship between germline *PALB2* mutation and melanoma risk. We have identified a missense mutation (chr16∶23647635, p.V78I) that incompletely segregates with disease in a family with cutaneous melanoma and breast cancer. We have also identified a further 7 previously reported missense variants for which the PALB2 protein function is undetermined. Genotyping of these variants did not reveal any significant differences in allele frequency between cases and controls, with the exception of one variant which occurred more frequently in controls (rs45568339). These data do not support a role for these rare PALB2 variants in melanoma susceptibility.

We also found a known deleterious mutation (rs118203998) causing a premature truncation of the protein (Y1183X) in an individual with four different cancer types, including melanoma. Neither this nor p.V78I variant has a population frequency reported in the 1000 Genomes Project or the ESP6500. Interestingly, the truncating mutation, p.Y1183X, has been reported by four previous studies. A report discusses three children with Fanconi anaemia that carried a *PALB2* p.Y1183X mutation. Each of these cases presented with an early childhood cancer at ages 0.7, 1.0 and 2.3 years with neuroblastoma, Wilms’ tumour and medulloblastoma respectively [5]. Rahman and colleagues also reported this mutation in three individuals with breast cancer [6]. Interestingly, in one of the families an individual with this genotype was diagnosed with melanoma at age 47, prior to developing breast cancer. In a third study, *PALB2* p.Y1183X was associated with breast cancer in a person presenting with two primary breast cancers. The mother of this proband was also a mutation carrier and had developed both breast cancer and pancreatic cancer [34]. Pancreatic and breast cancers have been previously associated with a *PALB2* mutation but this study was the first instance of it occurring in an individual with both cancer types. Lastly a population-based study of breast cancer predisposition by Tischowitz et al. found a family in which three cases had this genotype [8]. In our study we present an individual with two primary melanomas, bladder cancer, leukaemia and non-small cell lung cancer who is a carrier of the *PALB2* p.Y1183X variant. Taking into consideration that this specific mutation has been seen in one other individual with melanoma, this suggests that *PALB2* may play a role as a melanoma susceptibility gene and that this mutation could predispose to other cancers whose spectrum of disease extends beyond Fanconi anaemia, breast cancer or pancreatic cancer.

When we consider truncating mutations alone, as Rahman and colleagues did in relation to breast cancer susceptibility [6], the data overall are not strongly supportive of *PALB2* being a melanoma susceptibility gene. Larger sample sizes will clearly be needed to better characterize the spectrum of cancers associated with deleterious *PALB2* mutations along with a larger sample of melanoma-only families to unambiguously determine the role of *PALB2* in melanoma susceptibility.

## Supporting Information

Methods S1 Additional information on whole-genome and exome sequencing, Sanger sequencing, and iPLEX methods.
(DOCX)
