# Supplementary material for: Assessment of PALB2 as a Candidate Melanoma Susceptibility Gene
Source: PLoS One. 2014 Jun 20;9(6):e100683. doi: 10.1371/journal.pone.0100683 (PMC4065098; doi:10.1371/journal.pone.0100683)
Supplement: Methods S1 — Additional information on whole-genome and exome sequencing, Sanger sequencing, and iPLEX methods. (DOCX) [file pone.0100683.s001.docx]

**Methods S1**

Whole-genome and whole-exome sequencing

Whole-genome (n=12) and exome sequencing (n=25) was outsourced to Axeq Technologies in Seoul, South Korea. Sequencing was performed on the Illumina HiSeq 2000 platform using the Agilent SureSelect Human All Exon V4+UTRs enrichment kits. The data output was 100bp paired end reads with a median coverage of 96x. A further 3 genomes were sequenced using Complete Genomics. The sequencing data for each samples was mapped to the Human Genome build 19 (hg19) using the BWA alignment algorithm [[1](#_ENREF_1)]. SNPs and indels were detected using bcftools and SAMtools mpileup with disabled BAQ computation [[2](#_ENREF_2)]. Any variant that was found in dbSNP or the 1000 Genomes project was flagged to generate both a data set of novel mutations and also a data set of known mutations. Each sample had on average 90,000-100,000 variants compared to the human genome reference sequence. Variants were filtered for stringency using a quality score of >40, >2 alternate reads and >20% of all reads at a given position being the alternate read.

Sanger sequencing

Sanger sequencing was used to identify mutation events in *PALB2* in a cohort of familial melanoma cases. PCR reactions were run using ABI goldTaq PCR reagents. Standard manufacturer’s protocol was followed. Refer to Supplementary Table S1 for a list of the primers used.

Sequenom iPLEX

The *PALB2* variants were multiplexed using the Sequenom iPLEX gold system which allows up to 36 variants to be genotyped concurrently using 10ng of genomic DNA. The iPLEX Gold PCR amplification reactions were carried out in four 384-well plates according to supplier protocol and then transferred onto SpectroCHIP arrays using the MassARRAY Nanodispenser. The chips were then transferred to the Sequenom mass spectrometer where the data was generated. The results were analysed using the Typer Analyzer software 4.0.

**References**

1. Li H, Durbin R (2009) Fast and accurate short read alignment with Burrows-Wheeler transform. Bioinformatics 25: 1754-1760.

2. Li H, Handsaker B, Wysoker A, Fennell T, Ruan J, et al. (2009) The Sequence Alignment/Map format and SAMtools. Bioinformatics 25: 2078-2079.

**Table S1. Primers used for Sanger sequencing of *PALB2***

| **exon** | **forward primer** | **reverse primer** |
| --- | --- | --- |
| exon 1 | AACTGGGTCCCGGTGTCG | GCCTAAAACCCTGGGAAAGC |
| exon 2 & 3 | TGACTCCACCTTTCCACTTGC | AAGAACAATAGCCAAAATATACCTGGG |
| exon 4-1 | ATTCATCTGCCTGAATGAAATG | TGCTACTGATTTCTTCCTGTTCC |
| exon 4-2 | AGCTGCCAAGCAGAAGAAAG | ACCTTTAGGAGGAATGTGTTCAAG |
| exon 4-3 | CACCAGGGCGACTACAGTTC | AAGAGGAGAGGTTGCTTCCAG |
| exon 4-4 | CCCAGTGACACTCTTGATGG | AGGAAGTGCCAGGCAAATAG |
| exon 5-1 | TTGTCATCAGTGAAACAGATTGTC | GAAAGGCCCGTCTTTGTATG |
| exon 5-2 | AAACCAGTGGAGCCCTTTG | CACTTGCAGGGTGGTATGTG |
| exon 5-3 | GACTCAGTCTGTCTTGCCAGTG | AAGCAAGTCATGCTGTTTACATTC |
| exon 6 | AGTGGGTAATGCAGGCAGAC | GCCAATAGGTTGGCATAGAAAC |
| exon 7 | CCACAAAGCTCTTTCTTTTCACC | GCCTTGCATGGTCATAGCTC |
| exon 8 | GATTAAACAAAAATGAAACAACCAAGC | GGTTATTACCTGCACTTAAAACCAGC |
| exon 9 | TAATATTAAAAGGTTACTCCTCACATCACC | ACAGAAAAACGAGATCCTAGTTACCC |
| exon 10 | TCAACAATGCGGAGAAGGG | CTCTCTTATTTAATCTTCACAACAACCC |
| exon 11 | GGCAAAATTAACCCACAGTTC | ACTGCTTATGACTTACTGCTCTCAC |
| exon 12 | TCAGAGCCTATCGGTCATTGC | GCCTTTCAGAATGTCCCACC |
| exon 13-1 | CAATAGCCAACAGACCTCTAAGGC | TCATTTTAAGTGTCATTCAGATATTCTCC |
| exon 13-2 | AGGTTCCTGGAAGGTGACG | GCCATTTGAAGCTTTATGTACACC |
| M13 tag | TGTAAAACGACGGCCAGT | CAGGAAACAGCTATGACC |
